# Association between autoimmune thyroiditis and rheumatoid arthritis: a cross-sectional risk stratification study

**DOI:** 10.3389/fendo.2026.1764735

**Published:** 2026-04-01

**Authors:** Xiang Ji, Yina Zhang, Shilong Zhang, Shuai Shao, Jian Wu

**Affiliations:** 1 Center of Breast and Thyroid Surgery, Department of General Surgery, The Third People’s Hospital of Chengdu, Chengdu, Sichuan, China; 2 School of Medicine, Southwest Jiaotong University, Chengdu, Sichuan, China

**Keywords:** autoimmune thyroiditis, external validation, free triiodothyronine, NHANES, rheumatoid arthritis, uric acid

## Abstract

**Background:**

Autoimmune thyroiditis (AIT) frequently coexists with other autoimmune diseases, suggesting shared pathophysiological pathways. However, large-scale evidence supporting the relationship between AIT and rheumatoid arthritis (RA), as well as the potential metabolic and endocrine mediators linking the two, remains limited.

**Methods:**

A cross-sectional analysis was conducted using nationally representative data from NHANES 2007–2012 (n = 5,715). Multivariable logistic regression models were used to evaluate associations between AIT and RA. Mediation analyses assessed the potential indirect effects of uric acid and free triiodothyronine (FT3). A cross-sectional risk stratification nomogram for RA status among individuals with AIT was constructed. Model performance was assessed in NHANES and examined for consistency in an independent hospital-based clinical cohort from China (n = 196).

**Results:**

AIT was significantly associated with prevalent RA in the NHANES cohort after multivariable adjustment. Uric acid and FT3 were statistically associated with both AIT and RA in regression analyses. Exploratory mediation analyses suggested that uric acid accounted for a modest proportion of the observed association in NHANES (13.4%), with directionally consistent findings in the external clinical cohort, whereas FT3 demonstrated inconsistent and non-significant mediation across cohorts. The risk stratification model showed good discriminative performance in NHANES (AUC = 0.776) and maintained comparable discrimination in the external cohort (AUC = 0.724), with acceptable calibration.

**Conclusions:**

In this cross-sectional study, AIT was associated with prevalent RA, and selected metabolic and endocrine biomarkers were statistically related to this association. Although causal inference is precluded by the study design, these findings suggest the presence of shared metabolic and endocrine contexts underlying thyroid and joint autoimmunity and support further longitudinal investigation.

## 1 Introduction

Autoimmune diseases are a diverse group of chronic conditions marked by immune system dysregulation and the production of autoantibodies that target self-tissues (1). These disorders affect millions worldwide and often manifest with overlapping clinical and immunological characteristics. With increasing prevalence and the tendency for multiple autoimmune diseases to coexist in the same individual—a phenomenon known as polyautoimmunity—understanding the interactions among different autoimmune conditions has become a critical area of investigation (2). Such research has the potential to uncover shared pathophysiological pathways and guide the development of targeted interventions.

Autoimmune thyroiditis (AIT), particularly Hashimoto’s thyroiditis, is the most common organ-specific autoimmune disorder. It is characterized by thyroid dysfunction accompanied by the presence of thyroid peroxidase (TPOAb) and thyroglobulin antibodies (TgAb) (3). Rheumatoid arthritis (RA), in contrast, is a systemic autoimmune disease that primarily affects the synovial joints, leading to chronic inflammation and progressive joint destruction (4). While these diseases differ in their target tissues, they share many underlying features, including HLA associations, cytokine dysregulation, and hormonal influences—particularly in female-dominant populations (5). However, unlike the recent narrative review by Lichtiger et al., the present study provides original population-based evidence with external validation and quantitative analyses of the AIT–RA association and related metabolic/endocrine correlates. Previous observations suggest that patients with one autoimmune condition are at greater risk of developing another, yet the mechanisms driving this overlap remain insufficiently defined (6).

Despite case reports and small-scale studies noting the co-occurrence of AIT and RA, population-level evidence regarding their association remains limited. Notably, Yazdanifar et al. reported that thyroid dysfunction, anti-TPO positivity, and autoimmune thyroid disease were more common in patients with RA with greater disease severity, providing direct empirical support for a clinically relevant link between thyroid autoimmunity and RA (7). However, that study primarily focused on RA disease severity within a clinical RA population, whereas the present study extends the literature by evaluating the population-based association between AIT and prevalent RA, incorporating external validation, and further exploring shared metabolic and endocrine correlates.Even more scarce are data that explore the potential biological mediators linking the two conditions. Some experimental and clinical studies have implicated metabolic factors such as uric acid and endocrine regulators such as free triiodothyronine (FT3) in modulating immune responses (8, 9). These biomarkers may offer clues into the pathogenesis of autoimmune clustering, but their roles as mediators have not been thoroughly evaluated.

To address this gap, we conducted a cross-sectional study using data from the National Health and Nutrition Examination Survey (NHANES) 2007–2012, a nationally representative dataset rich in demographic, clinical, and laboratory variables (10, 11). We examined the relationship between AIT and RA in U.S. adults, identifying key biomarkers associated with both conditions.

We further explored whether uric acid and FT3 mediated the relationship between AIT and RA through formal mediation analysis. Lastly, we constructed a multivariable logistic regression model to identify individuals with RA risk among those with AIT, presenting the results using a nomogram and ROC analysis.

By investigating these relationships within a large, well-characterized population, this study aims to improve our understanding of autoimmune disease clustering and identify metabolic and endocrine pathways that may contribute to shared pathophysiology. These findings may support future research into risk stratification and early intervention strategies for polyautoimmune patients.

## 2 Methods

### 2.1 Study design and population

This study included a discovery cohort from NHANES and an independent external validation cohort from a tertiary hospital in China.

**Figure 1:**
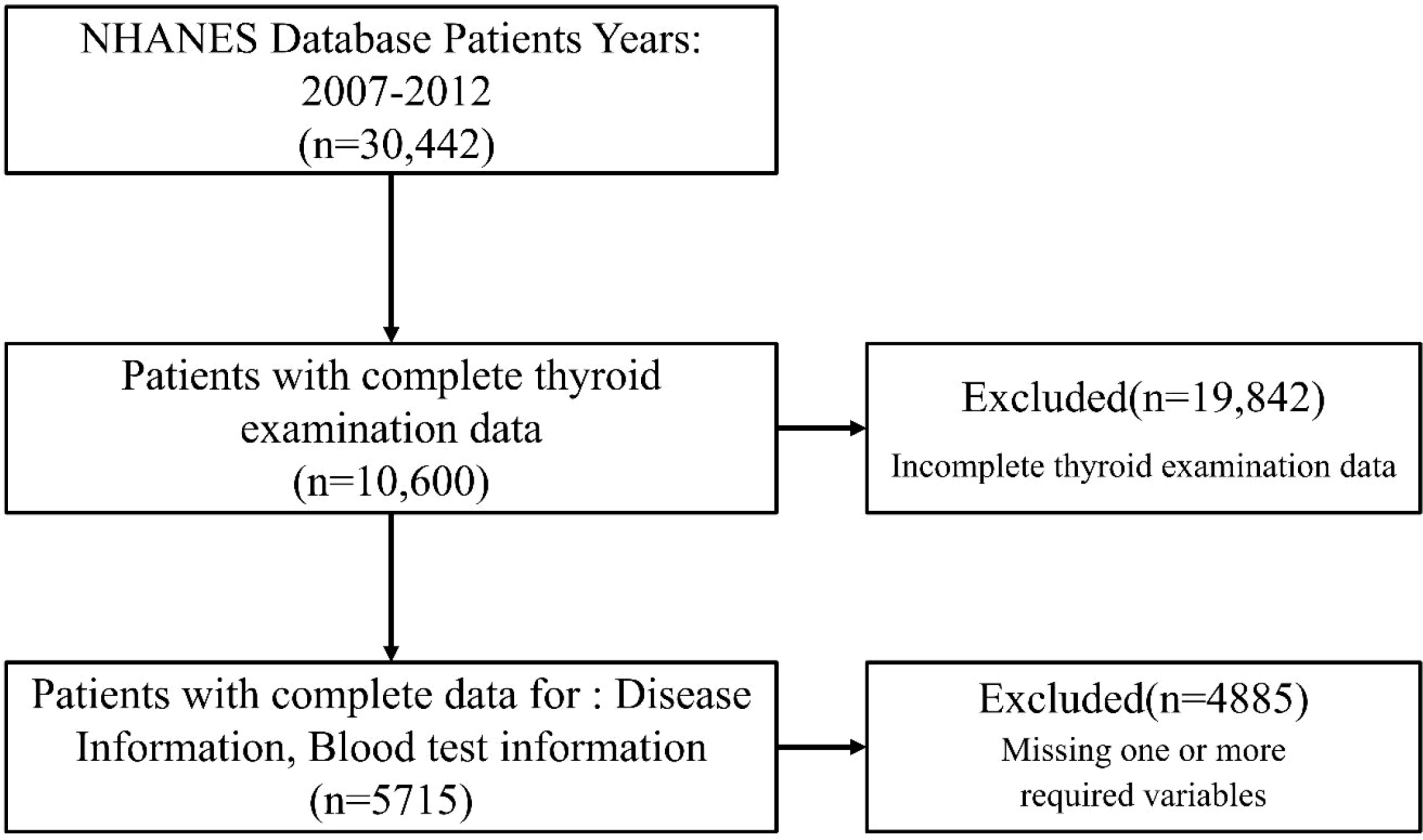
Flowchart of participant selection from the NHANES 2007–2012 dataset.

We analyzed publicly available data from the 2007–2012 NHANES cycles, which applied a multistage, stratified sampling design to ensure representativeness. All participants provided written informed consent, and data were de-identified. Among 30,442 individuals screened, those aged ≥18 years with complete thyroid function markers (TSH, FT3, FT4, TPOAb, TgAb), confirmed diagnostic information for autoimmune thyroiditis (AIT) and rheumatoid arthritis (RA), and essential clinical variables were selected. After excluding participants with missing data, 5,715 adults were included in the final analysis (**Figure 1**). The flowchart of participant selection for the external validation cohort is presented in **Supplementary Figure 1**.

To confirm the robustness of findings, we prospectively recruited 196 adult patients from the Department of Breast and Thyroid Surgery at the Third People’s Hospital of Chengdu between January 2023 and June 2024. Inclusion required complete thyroid evaluation and confirmed AIT diagnosis; exclusions included pregnancy, major organ dysfunction, malignancy, and severe autoimmune comorbidities. Cases comprised 98 AIT patients and 98 non-AIT controls. Diagnostic criteria aligned with NHANES definitions (TPOAb ≥ 9 IU/mL and/or TgAb ≥ 4 IU/mL; RA based on 2010 ACR/EULAR criteria). Written informed consent was obtained, and ethical approval was granted by the institutional review board.

### 2.2 Exposure variable: thyroid autoimmunity

In accordance with prior NHANES-based epidemiological studies and data availability, thyroid autoimmunity was defined by the presence of thyroid peroxidase antibody (TPOAb ≥9 IU/mL) and/or thyroglobulin antibody (TgAb ≥4 IU/mL). Owing to the lack of standardized thyroid ultrasound findings or clinical diagnostic information for thyroiditis in NHANES, antibody positivity was used as a surrogate marker of autoimmune thyroid involvement rather than clinically confirmed autoimmune thyroiditis.

### 2.3 Outcome variable: rheumatoid arthritis

Rheumatoid arthritis was determined based on participants’ responses to the questionnaire item asking whether a doctor had ever diagnosed them with the condition. Individuals who answered “yes” were classified as having rheumatoid arthritis, while those who answered “no” or had missing responses were excluded from the analysis.

### 2.4 Covariates

A broad set of covariates was included to characterize the demographic, clinical, and biochemical context of autoimmune thyroiditis and rheumatoid arthritis within the NHANES population. Covariate selection was guided by clinical relevance to autoimmune and metabolic conditions, as well as data availability in NHANES. All variables were derived from the publicly released NHANES data dictionary and were collected using standardized questionnaires, physical examinations, and laboratory procedures following quality control protocols established by the Centers for Disease Control and Prevention.

Demographic variables included age (continuous) and sex (male or female). Although race/ethnicity and socioeconomic indicators (such as education level and poverty income ratio) are available in NHANES, they were not included in the current analysis to maintain model parsimony and focus on clinical and biochemical features.

Self-reported physician-diagnosed comorbidities were included to describe systemic health status potentially relevant to autoimmune conditions, including diabetes, hypertension, congestive heart failure, coronary heart disease, angina pectoris, other heart disease, stroke, asthma, emphysema, chronic bronchitis, renal failure, kidney stones, liver disease, gout, malignant neoplasm, sleep disorder, and rheumatoid arthritis.

Psychological status was assessed using nine depressive symptoms from the NHANES depression questionnaire (DPQ), including reduced interest, depressed mood, sleep disturbance, fatigue, appetite change, low self-worth, difficulty concentrating, psychomotor changes, and suicidal ideation. These variables have been widely used in prior NHANES-based studies to capture mental health–related factors.

Laboratory variables encompassed multiple physiological systems and were grouped according to established biomarker frameworks for autoimmune and metabolic research:

(1) Thyroid-related markers: free thyroxine, thyroid-stimulating hormone, total and free triiodothyronine, total thyroxine, thyroglobulin, thyroglobulin antibody, and thyroid peroxidase antibody;
(2) Liver function: alanine aminotransferase, aspartate aminotransferase, alkaline phosphatase, gamma-glutamyltransferase, lactate dehydrogenase, and total bilirubin;
(3) Renal function: creatinine, blood urea nitrogen, and uric acid;
(4) Lipid metabolism: triglycerides, total cholesterol, and high-density lipoprotein cholesterol;
(5) Glucose metabolism: serum glucose and glycated hemoglobin;
(6) Electrolytes: calcium, phosphorus, chloride, potassium, sodium, and bicarbonate;
(7) Protein and nutritional status: albumin, total protein, and globulin;
(8) Inflammatory marker: C-reactive protein;
(9) Hematological indices: white blood cell count, red blood cell count, hemoglobin, hematocrit, mean corpuscular volume, mean corpuscular hemoglobin, mean corpuscular hemoglobin concentration, red cell distribution width, platelet count, and mean platelet volume.

Additional biochemical measures included micronutrients and environmental exposure indicators, such as serum and red blood cell folate, pyridoxal 5’-phosphate, 4-pyridoxic acid, vitamin D metabolites, serum iron, blood cadmium, blood lead, mercury species, and serum osmolality.

These covariates have been extensively used in prior NHANES-based epidemiological studies and provide a comprehensive overview of systemic clinical and biochemical characteristics relevant to autoimmune disease research.

### 2.5 Internal validation and nomogram development

Internal validation was conducted in the NHANES cohort to assess model optimism and stability using bootstrap resampling (B = 2,000). Optimism-corrected C-statistics and calibration slopes were calculated using the rms package in R, with optimism defined as the difference between apparent and corrected performance. A calibration slope close to 1 indicates good agreement between predicted and observed values. In addition, 95% confidence intervals for the AUC were estimated using the pROC package with 2,000 bootstrap resamples.

Variables included in the final nomogram were selected based on statistical significance in multivariable logistic regression (P < 0.05) and their association with both autoimmune thyroiditis and rheumatoid arthritis.

### 2.6 Statistical analysis

All analyses were performed using R software (version 4.3.0). For NHANES, the complex sampling design was accounted for by incorporating sampling weights, stratification, and clustering using the “survey” package, ensuring nationally representative estimates. Continuous variables were presented as weighted means ± standard deviations or medians with interquartile ranges, and categorical variables as weighted percentages. Group comparisons were conducted using design-based t-tests or the Mann–Whitney U test for continuous variables, and the Rao–Scott χ² test for categorical variables. Univariate logistic regression was used to explore associations between individual biomarkers/comorbidities and autoimmune thyroiditis or rheumatoid arthritis. Variables with P < 0.1 in univariate analyses were entered into multivariable models to examine their joint associations, with adjusted odds ratios (aORs) and 95% confidence intervals (CIs) reported. A two-sided P < 0.05 was considered significant. To explore shared statistical associations, biomarkers simultaneously associated with both conditions were selected for exploratory mediation analyses using the “mediation” package (5,000 bootstrap iterations).

In the clinical validation cohort, we replicated the mediation analysis workflow and constructed a logistic regression-based RA classification model. Discrimination was evaluated by ROC curves and the area under the curve (AUC), while calibration was assessed using calibration plots, Brier scores, and the Hosmer–Lemeshow goodness-of-fit test. Missing data were assumed missing at random and handled using multivariate imputation by chained equations (“mice” package). Structural equation modeling (SEM) using the “lavaan” package was conducted as a sensitivity analysis to assess the robustness of the mediation findings obtained from the primary bootstrap-based approach. As the direction and statistical significance of indirect effects were consistent across methods, SEM results are not reported separately to avoid redundancy. All procedures followed official NHANES analytical guidelines.

## 3 Results

### 3.1 Participant characteristics

A total of 5,715 participants were included in the analysis, of whom 545 (9.5%) were classified as having autoimmune thyroiditis and 5,170 as non–autoimmune thyroiditis individuals. Baseline characteristics of the two groups are summarized in **Table 1**. Baseline characteristics of the external validation cohort are presented in **Supplementary Table 1**, and a comparison between the external validation cohort and the NHANES database cohort is shown in **Supplementary Table 2**.

**Table 1:** Baseline characteristics of participants according to autoimmune thyroiditis status.

Variable	Normal (n=5170)	Autoimmune thyroiditis (n=545)	P
Gender (%)			<0.001
Male	2677 (51.8)	174 (31.9)	
Female	2493 (48.2)	371 (68.1)	
Diabetes (%)	730 (14.1)	84 (15.4)	0.449
Hypertension (%)	1850 (35.8)	213 (39.1)	0.139
Congestive Heart Failure (%)	185 (3.6)	20 (3.7)	1
Coronary Heart Disease (%)	226 (4.4)	28 (5.1)	0.474
Angina Pectoris (%)	148 (2.9)	16 (2.9)	1
Heart Disease (%)	246 (4.8)	27 (5.0)	0.922
Stroke (%)	207 (4.0)	24 (4.4)	0.737
Asthma (%)	707 (13.7)	74 (13.6)	1
Emphysema (%)	153 (3.0)	17 (3.1)	0.939
Chronic Bronchitis (%)	319 (6.2)	42 (7.7)	0.192
Renal Failure (%)	163 (3.2)	9 (1.7)	0.069
Kidney Stones (%)	529 (10.2)	51 (9.4)	0.571
Rheumatoid Arthritis (%)	1483 (28.7)	181 (33.2)	0.031
Liver Disease (%)	168 (3.2)	18 (3.3)	1
Gout (%)	273 (5.3)	23 (4.2)	0.337
Malignant Neoplasm (%)	536 (10.4)	65 (11.9)	0.291
Sleep Disorder (%)	413 (8.0)	39 (7.2)	0.548
Little Interest in Doing Things (%)	867 (18.2)	92 (18.4)	0.968
Feeling Down, Depressed, or Hopeless (%)	901 (18.9)	101 (20.1)	0.575
Trouble Sleeping or Sleeping Too Much (%)	1214 (28.1)	131 (30.0)	0.451
Feeling Tired or Having Little Energy (%)	1684 (39.1)	172 (38.4)	0.813
Poor Appetite or Overeating (%)	780 (16.6)	77 (15.5)	0.591
Feeling Bad About Yourself (%)	632 (13.0)	74 (14.4)	0.411
Trouble Concentrating on Things (%)	614 (12.6)	49 (9.6)	0.065
Moving or Speaking Slowly or Too Fast (%)	393 (7.9)	43 (8.2)	0.847
Thoughts That You Would Be Better Off Dead (%)	152 (3.0)	7 (1.3)	0.036
Age (mean (SD))	50.04 (17.80)	55.62 (16.87)	<0.001
Alanine Aminotransferase ALT (U/L) (median [IQR])	21.00 [17.00, 29.00]*	21.00 [17.00, 27.00]*	0.792
Aspartate Aminotransferase AST (U/L) (median [IQR])	24.00 [20.00, 28.00]*	24.00 [20.00, 28.00]*	0.266
Alkaline Phosphatase (U/L) (median [IQR])	67.00 [55.00, 81.00]*	67.00 [55.00, 81.00]*	0.611
Gamma-Glutamyltransferase (U/L) (median [IQR])	21.00 [15.00, 32.75]*	19.00 [14.00, 30.00]*	0.001
Lactate Dehydrogenase (U/L) (median [IQR])	129.00 [115.00, 146.00]*	132.00 [117.00, 152.00]*	0.010
Total Bilirubin (mg/dL) (mean (SD))	0.77 (0.30)	0.76 (0.26)	0.254
Total Bilirubin (umol/L) (mean (SD))	13.19 (5.19)	12.93 (4.50)	0.254
Creatinine (mg/dL) (median [IQR])	0.84 [0.72, 1.02]*	0.82 [0.70, 0.94]*	0.001
Creatinine (mol/L) (median [IQR])	74.26 [63.65, 90.17]*	72.49 [61.88, 83.10]*	0.001
Blood Urea Nitrogen (mmol/L) (median [IQR])	4.28 [3.57, 5.71]*	4.64 [3.57, 5.71]*	0.001
Uric Acid (umol/L) (mean (SD))	330.52 (86.74)	318.82 (84.79)	0.003
Triglycerides (mmol/L) (median [IQR])	1.45 [0.95, 2.22]*	1.47 [0.97, 2.21]*	0.482
Cholesterol (mmol/L) (mean (SD))	5.08 (1.08)	5.20 (1.14)	0.014
Direct HDL-Cholesterol (mmol/L) (mean (SD))	1.34 (0.41)	1.40 (0.43)	0.001
Total Cholesterol (mmol/L) (mean (SD))	5.07 (1.07)	5.18 (1.12)	0.022
Serum Glucose (mmol/L) (median [IQR])	5.16 [4.72, 5.82]*	5.27 [4.83, 5.94]*	0.001
Glycated Hemoglobin (%) (median [IQR])	5.50 [5.20, 5.90]*	5.60 [5.30, 6.00]*	<0.001
Total Calcium (mmol/L) (mean (SD))	2.35 (0.09)	2.35 (0.10)	0.937
Phosphorus (mg/dL) (mean (SD))	3.74 (0.57)	3.78 (0.54)	0.132
Phosphorus (mmol/L) (mean (SD))	1.21 (0.18)	1.22 (0.18)	0.132
Chloride (mmol/L) (mean (SD))	104.01 (2.90)	103.90 (2.99)	0.370
Potassium (mmol/L) (mean (SD))	3.98 (0.34)	3.97 (0.33)	0.616
Sodium (mmol/L) (mean (SD))	139.31 (2.34)	139.20 (2.43)	0.273
Bicarbonate (mmol/L) (mean (SD))	25.04 (2.28)	25.14 (2.25)	0.363
Albumin (g/dL) (mean (SD))	4.23 (0.33)	4.20 (0.36)	0.035
Total Protein (g/dL) (mean (SD))	7.17 (0.47)	7.17 (0.48)	0.982
Globulin (g/dL) (mean (SD))	2.94 (0.47)	2.98 (0.44)	0.120
C-Reactive Protein (mg/dL) (median [IQR])	0.20 [0.08, 0.47]*	0.18 [0.08, 0.41]*	0.137
Free Thyroxine (ng/dL) (median [IQR])	0.80 [0.70, 0.90]*	0.80 [0.70, 0.90]*	0.088
Thyroid Stimulating Hormone (uIU/mL) (median [IQR])	1.53 [1.03, 2.27]*	2.41 [1.42, 3.92]*	<0.001
Triiodothyronine (T3), Total (ng/dL) (median [IQR])	111.00 [98.00, 125.00]*	106.00 [91.00, 121.00]*	<0.001
Triiodothyronine (T3), Free (pg/mL) (median [IQR])	3.10 [2.90, 3.40]*	3.00 [2.80, 3.30]*	<0.001
Total Thyroxine (T4) (ug/dL) (mean (SD))	7.92 (1.62)	7.97 (2.08)	0.479
Thyroglobulin (ng/mL) (median [IQR])	10.27 [6.02, 17.35]*	4.04 [0.65, 18.39]*	<0.001
Thyroglobulin Antibody (IU/mL) (median [IQR])	0.60 [0.60, 0.60]*	8.80 [0.90, 46.10]*	<0.001
Thyroid Peroxidase Antibody (IU/mL) (median [IQR])	0.60 [0.30, 1.20]*	112.80 [32.20, 305.30]*	<0.001
White Blood Cell Count (1000 cells/uL) (median [IQR])	7.00 [5.70, 8.40]*	6.80 [5.60, 8.20]*	0.079
Red Blood Cell Count (million cells/uL) (mean (SD))	4.67 (0.50)	4.55 (0.47)	<0.001
Hemoglobin (g/dL) (mean (SD))	14.25 (1.54)	13.94 (1.51)	<0.001
Hematocrit (%) (mean (SD))	41.44 (4.26)	40.54 (4.23)	<0.001
Mean Cell Volume (fL) (mean (SD))	88.92 (5.49)	89.38 (5.13)	0.064
Mean Cell Hemoglobin (pg) (mean (SD))	30.57 (2.27)	30.74 (2.08)	0.105
Mean Cell Hemoglobin Concentration (g/dL) (mean (SD))	34.36 (0.96)	34.38 (0.88)	0.656
Red Cell Distribution Width (%) (median [IQR])	12.60 [12.20, 13.30]*	12.60 [12.20, 13.40]*	0.718
Platelet Count (1000 cells/uL) (mean (SD))	258.04 (67.27)	252.64 (61.83)	0.072
Mean Platelet Volume (fL) (mean (SD))	7.80 (0.85)	7.82 (0.80)	0.573
Lymphocyte Percent (%) (mean (SD))	30.48 (8.67)	29.80 (8.04)	0.082
Monocyte Percent (%) (median [IQR])	7.70 [6.20, 9.10]*	7.60 [6.40, 9.10]*	0.517
Segmented Neutrophil Percent (%) (mean (SD))	58.10 (9.73)	58.72 (9.00)	0.155
Eosinophil Percent (%) (median [IQR])	2.30 [1.60, 3.70]*	2.50 [1.70, 3.70]*	0.085
Basophil Percent (%) (median [IQR])	0.60 [0.40, 0.80]*	0.60 [0.40, 0.90]*	0.157
Lymphocyte Number (1000/uL) (median [IQR])	2.00 [1.63, 2.50]*	2.00 [1.60, 2.50]*	0.013
Monocyte Number (1000/uL) (median [IQR])	0.50 [0.40, 0.60]*	0.50 [0.40, 0.60]*	0.192
Segmented Neutrophil Number (1000 cells/uL) (median [IQR])	4.00 [3.10, 5.20]*	3.90 [3.10, 5.00]*	0.391
Eosinophil Number (1000/uL) (median [IQR])	0.20 [0.10, 0.30]*	0.20 [0.10, 0.30]*	0.633
Basophil Number (1000 cells/uL) (median [IQR])	0.00 [0.00, 0.10]*	0.00 [0.00, 0.10]*	0.547
Serum Iron (ug/dL) (mean (SD))	85.31 (35.12)	83.13 (33.76)	0.168
Serum Iron (ug/L) (mean (SD))	15.28 (6.29)	14.89 (6.05)	0.169
Blood Cadmium (ug/L) (median [IQR])	0.35 [0.22, 0.62]*	0.37 [0.25, 0.58]*	0.160
Blood Cadmium (nmol/L) (median [IQR])	3.11 [1.96, 5.52]*	3.29 [2.22, 5.16]*	0.160
Blood Lead (ug/dL) (median [IQR])	1.40 [0.94, 2.20]*	1.38 [0.89, 2.18]*	0.211
Blood Lead (umol/L) (median [IQR])	0.07 [0.04, 0.11]*	0.07 [0.04, 0.10]*	0.210
Total Mercury (ug/L) (median [IQR])	0.89 [0.49, 1.67]*	0.83 [0.45, 1.60]*	0.202
Total Mercury (umol/L) (median [IQR])	4.40 [2.40, 8.30]*	4.10 [2.20, 8.00]*	0.199
Inorganic Mercury (ug/L) (median [IQR])	0.25 [0.25, 0.25]*	0.25 [0.25, 0.35]*	0.228
Inorganic Mercury (umol/L) (median [IQR])	1.25 [1.25, 1.25]*	1.25 [1.25, 1.75]*	0.228
Serum Folate (nmol/L) (median [IQR])	36.10 [24.10, 54.20]*	41.50 [27.50, 61.40]*	<0.001
Red Blood Cell Folate (nmol/L) (mean (SD))	1,197.27 (568.78)	1,329.42 (671.07)	<0.001
Pyridoxal 5’-Phosphate (nmol/L) (median [IQR])	44.60 [27.00, 76.00]*	44.30 [27.00, 80.00]*	0.594
4-Pyridoxic Acid (nmol/L) (median [IQR])	24.50 [15.70, 47.98]*	26.70 [17.50, 59.80]*	0.001
25-Hydroxyvitamin D2+D3 (nmol/L) (mean (SD))	62.16 (25.59)	65.52 (26.06)	0.004
25-Hydroxyvitamin D2 (nmol/L) (median [IQR])	1.45 [1.45, 1.45]*	1.45 [1.45, 1.45]*	0.646
25-Hydroxyvitamin D3 (nmol/L) (mean (SD))	58.25 (25.22)	61.49 (24.82)	0.004
Osmolality (mmol/Kg) (mean (SD))	278.53 (5.18)	278.73 (5.22)	0.409

* Non-normally distributed variables are presented as median (interquartile range) and were compared using the Mann–Whitney U test.

With respect to demographic characteristics, participants with autoimmune thyroiditis were more likely to be female (68.1% vs. 48.2%, *P* < 0.001) and were significantly older than those without autoimmune thyroiditis (mean age: 55.62 ± 16.87 vs. 50.04 ± 17.80 years, *P* < 0.001).

In terms of comorbidities, the prevalence of rheumatoid arthritis was higher among individuals with autoimmune thyroiditis (33.2% vs. 28.7%, *P* = 0.031). Differences in the prevalence of chronic bronchitis and renal dysfunction did not reach statistical significance (*P* = 0.19 and *P* = 0.069, respectively). No significant differences were observed for other chronic conditions, including diabetes mellitus, hypertension, cardiovascular disease, stroke, liver disease, gout, malignancy, or sleep disorders.

Regarding mental health–related variables, the distribution of depressive symptoms—including loss of interest, depressed mood, sleep disturbance, fatigue, appetite changes, impaired concentration, psychomotor changes, and suicidal ideation—was comparable between groups, with no statistically significant differences observed (all *P* > 0.5).

Laboratory parameters demonstrated several between-group differences. Individuals with autoimmune thyroiditis exhibited lower serum uric acid levels and modest alterations in renal-related markers, while differences in creatinine did not reach statistical significance. Lipid profiles showed slightly higher levels of direct high-density lipoprotein cholesterol and total cholesterol in the autoimmune thyroiditis group. As expected, thyroid-related markers—including thyroid-stimulating hormone, thyroglobulin antibody, and thyroid peroxidase antibody—were markedly elevated among participants with autoimmune thyroiditis (all *P* < 0.001). In hematologic measures, red blood cell count, hemoglobin, and hematocrit were significantly lower in the autoimmune thyroiditis group, whereas C-reactive protein levels did not differ significantly between the two groups.

### 3.2 Association between comorbid conditions and autoimmune thyroiditis

**Figure 2:**
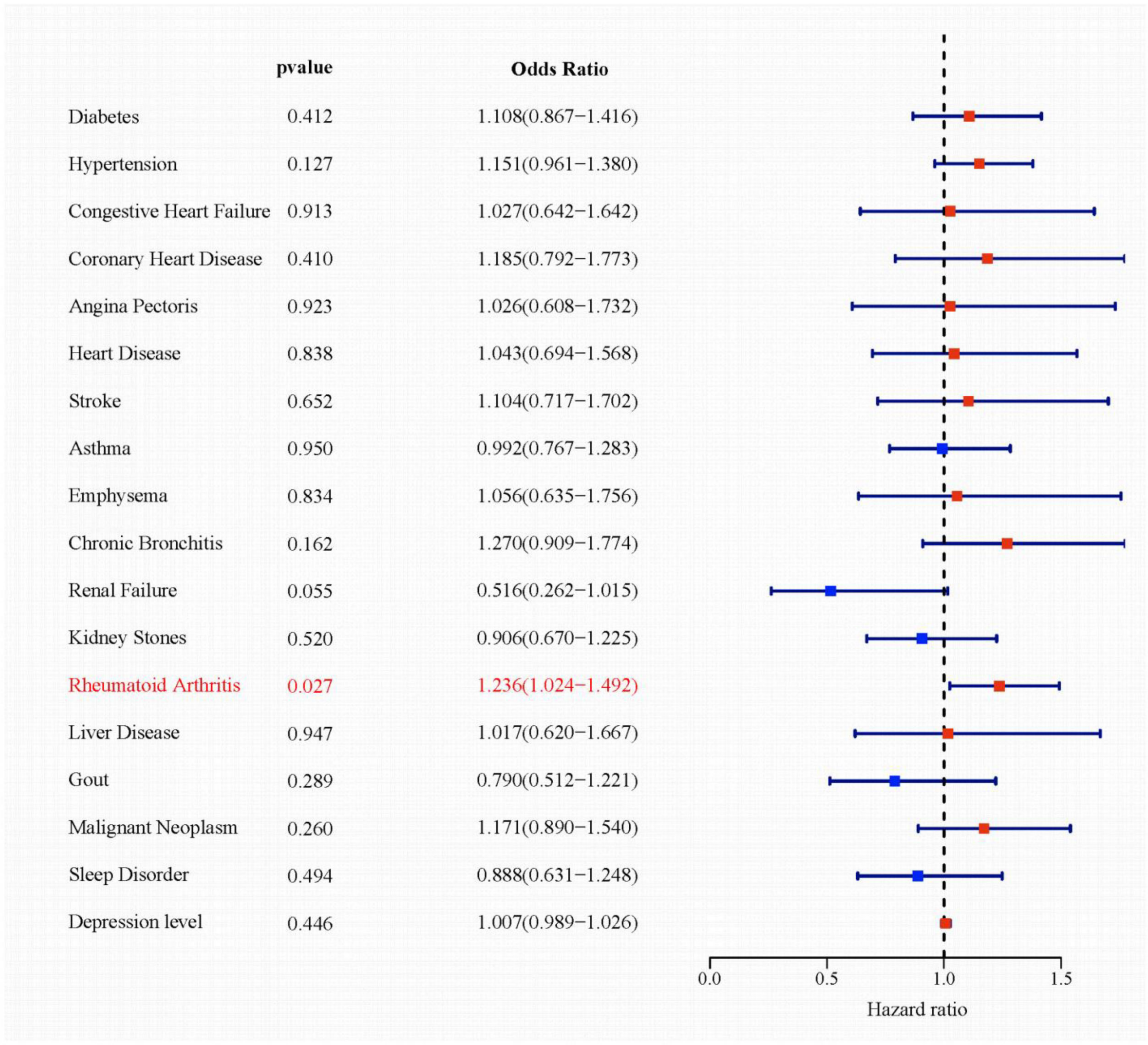
Forest plot of the associations between comorbid conditions and AIT.

Univariate logistic regression analysis was conducted to evaluate the associations between comorbidities and autoimmune thyroiditis (**Figure 2**). Rheumatoid arthritis emerged as the only comorbidity significantly associated with autoimmune thyroiditis (OR = 1.236, 95% CI: 1.024–1.492, P = 0.027). Although renal failure approached statistical significance (P = 0.055), it did not meet the conventional threshold. Other conditions, including diabetes, hypertension, chronic bronchitis, gout, and malignancy, showed no significant associations (all P > 0.05). These results indicate a specific statistical association between rheumatoid arthritis and autoimmune thyroiditis and motivated further exploratory analyses to examine potential shared metabolic and endocrine correlates. To address potential confounding by gout and urate-related conditions, a sensitivity analysis excluding participants with gout was performed. The association between AIT and RA, as well as the direction and significance of key mediating biomarkers, remained materially unchanged (**Supplementary Table 3**, **S4**).

### 3.3 Analysis of biomarkers associated with autoimmune thyroiditis

**Figure 3:**
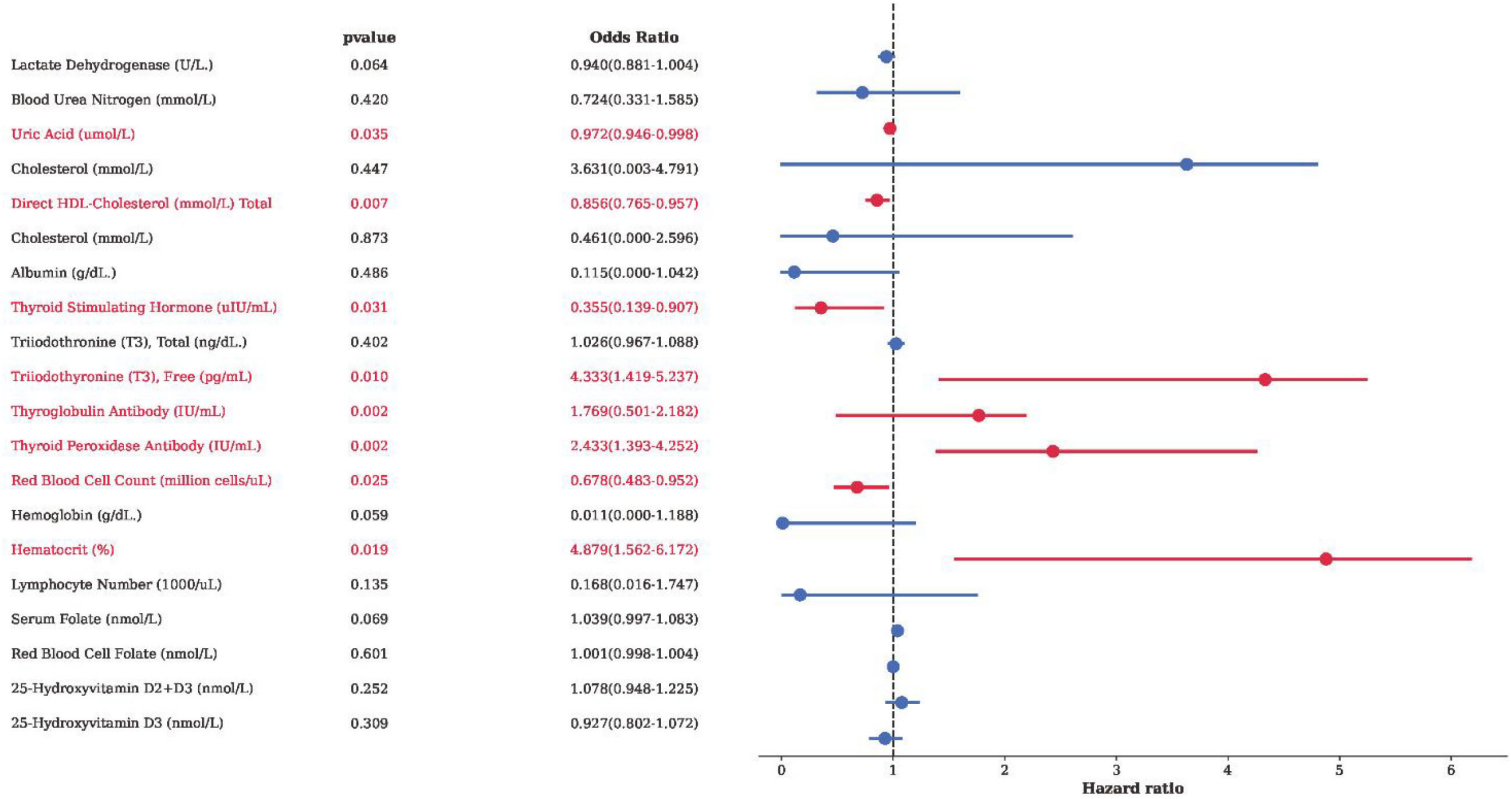
Forest plot of the associations between biochemical and immunological indicators and AIT.

Univariate and multivariable logistic regression analyses were performed to examine associations between circulating blood biomarkers and autoimmune thyroiditis (**Figure 3** and **Table 2**).

**Table 2:** Association between blood biochemical indicators and AIT: univariate logistic regression analysis.

Blood test indicators	OR	CI_lower	CI_upper	P_value
Alanine Aminotransferase ALT (U/L)	1.000	0.995	1.004	0.859
Aspartate Aminotransferase AST (U/L)	1.003	0.997	1.008	0.347
Alkaline Phosphatase (U/L)	1.001	0.997	1.004	0.728
Gamma-Glutamyltransferase (U/L)	0.997	0.993	1.000	0.057
Lactate Dehydrogenase (U/L)	1.003	1.001	1.006	0.015
Total Bilirubin (mg/dL)	0.837	0.617	1.136	0.254
Creatinine (mg/dL)	0.742	0.538	1.024	0.069
Blood Urea Nitrogen (mmol/L)	1.048	1.010	1.087	0.013
Uric Acid (umol/L)	0.998	0.997	0.999	0.003
Triglycerides (mmol/L)	0.993	0.931	1.060	0.843
Cholesterol (mmol/L)	1.105	1.020	1.196	0.014
Direct HDL-Cholesterol (mmol/L)	1.393	1.138	1.704	0.001
Total Cholesterol (mmol/L)	1.099	1.014	1.191	0.022
Serum Glucose (mmol/L)	1.026	0.989	1.065	0.172
Glycated Hemoglobin (%)	1.070	0.992	1.155	0.079
Total Calcium (mmol/L)	0.963	0.375	2.469	0.937
Phosphorus (mg/dL)	1.126	0.965	1.313	0.132
Chloride (mmol/L)	0.986	0.957	1.016	0.370
Potassium (mmol/L)	0.935	0.720	1.215	0.616
Sodium (mmol/L)	0.980	0.944	1.016	0.273
Bicarbonate (mmol/L)	1.018	0.979	1.058	0.363
Albumin (g/dL)	0.757	0.585	0.980	0.035
Total Protein (g/dL)	1.002	0.832	1.207	0.982
Globulin (g/dL)	1.158	0.963	1.392	0.120
C-Reactive Protein (mg/dL)	0.966	0.867	1.077	0.537
Free Thyroxine (ng/dL)	1.285	0.826	1.999	0.266
Thyroid Stimulating Hormone (uIU/mL)	1.335	1.272	1.401	0.000
Triiodothyronine (T3), Total (ng/dL)	0.994	0.990	0.998	0.002
Triiodothyronine (T3), Free (pg/mL)	0.638	0.505	0.805	0.000
Total Thyroxine (T4) (ug/dL)	1.019	0.967	1.073	0.479
Thyroglobulin (ng/mL)	0.999	0.996	1.002	0.495
Thyroglobulin Antibody (IU/mL)	1.658	1.573	1.749	0.000
Thyroid Peroxidase Antibody (IU/mL)	1.110	1.099	1.122	0.000
White Blood Cell Count (1000 cells/uL)	0.963	0.923	1.004	0.078
Red Blood Cell Count (million cells/uL)	0.601	0.503	0.718	0.000
Hemoglobin (g/dL)	0.882	0.833	0.933	0.000
Hematocrit (%)	0.952	0.933	0.972	0.000
Mean Cell Volume (fL)	1.016	0.999	1.033	0.064
Mean Cell Hemoglobin (pg)	1.033	0.993	1.075	0.104
Mean Cell Hemoglobin Concentration (g/dL)	1.021	0.931	1.121	0.656
Red Cell Distribution Width (%)	1.026	0.960	1.098	0.446
Platelet Count (1000 cells/uL)	0.999	0.997	1.000	0.072
Mean Platelet Volume (fL)	1.030	0.929	1.143	0.573
Lymphocyte Percent (%)	0.991	0.980	1.001	0.082
Monocyte Percent (%)	1.008	0.973	1.045	0.651
Segmented Neutrophil Percent (%)	1.007	0.997	1.016	0.155
Eosinophil Percent (%)	1.003	0.965	1.042	0.890
Basophil Percent (%)	0.991	0.826	1.190	0.926
Lymphocyte Number (1000/uL)	0.849	0.749	0.962	0.010
Monocyte Number (1000/uL)	0.828	0.516	1.329	0.434
Segmented Neutrophil Number (1000 cells/uL)	0.980	0.931	1.032	0.441
Eosinophil Number (1000/uL)	0.838	0.499	1.408	0.504
Basophil Number (1000 cells/uL)	0.421	0.085	2.078	0.288
Serum Iron (ug/dL)	0.998	0.996	1.001	0.168
Serum Iron (ug/L)	0.990	0.976	1.004	0.169
Blood Cadmium (ug/L)	0.939	0.806	1.094	0.417
Blood Cadmium (nmol/L)	0.993	0.976	1.010	0.416
Blood Lead (ug/dL)	0.970	0.912	1.032	0.337
Total Mercury (ug/L)	0.988	0.945	1.032	0.577
Inorganic Mercury (ug/L)	1.213	0.801	1.838	0.362
Serum Folate (nmol/L)	1.005	1.003	1.008	0.000
Red Blood Cell Folate (nmol/L)	1.000	1.000	1.000	0.000
Pyridoxal 5’-Phosphate (nmol/L)	1.000	0.999	1.001	0.450
4-Pyridoxic Acid (nmol/L)	1.000	1.000	1.000	0.634
25-Hydroxyvitamin D2+D3 (nmol/L)	1.005	1.002	1.008	0.004
25-Hydroxyvitamin D2 (nmol/L)	1.001	0.992	1.011	0.797
25-Hydroxyvitamin D3 (nmol/L)	1.005	1.002	1.008	0.004
Osmolality (mmol/Kg)	1.007	0.990	1.025	0.408

In univariate analyses, several metabolic and hematologic markers were statistically associated with autoimmune thyroiditis. Lower serum uric acid levels and higher levels of direct high-density lipoprotein cholesterol and total cholesterol were associated with autoimmune thyroiditis (all *P* < 0.05). Erythroid indices, including red blood cell count, hemoglobin, and hematocrit, were inversely associated with autoimmune thyroiditis.

Thyroid-related biomarkers demonstrated the strongest associations. Thyroid-stimulating hormone, thyroglobulin antibody, and thyroid peroxidase antibody were positively associated with autoimmune thyroiditis, whereas free triiodothyronine was inversely associated in univariate analysis (all *P* < 0.001).

In multivariable models adjusting for potential confounders, several biomarkers remained independently associated with autoimmune thyroiditis. Thyroglobulin antibody and thyroid peroxidase antibody showed robust positive associations, while free triiodothyronine and thyroid-stimulating hormone remained inversely associated. In addition, serum uric acid and direct high-density lipoprotein cholesterol retained statistically significant associations after adjustment. Selected hematologic parameters, including red blood cell count and hematocrit, also remained associated with autoimmune thyroiditis in adjusted analyses.

Collectively, these findings highlight a pattern of concurrent metabolic, hematologic, and thyroid-related alterations associated with autoimmune thyroiditis. Given the cross-sectional nature of the study, these associations should be interpreted as exploratory and descriptive rather than causal.

### 3.4 Analysis of biomarkers associated with rheumatoid arthritis

**Figure 4:**
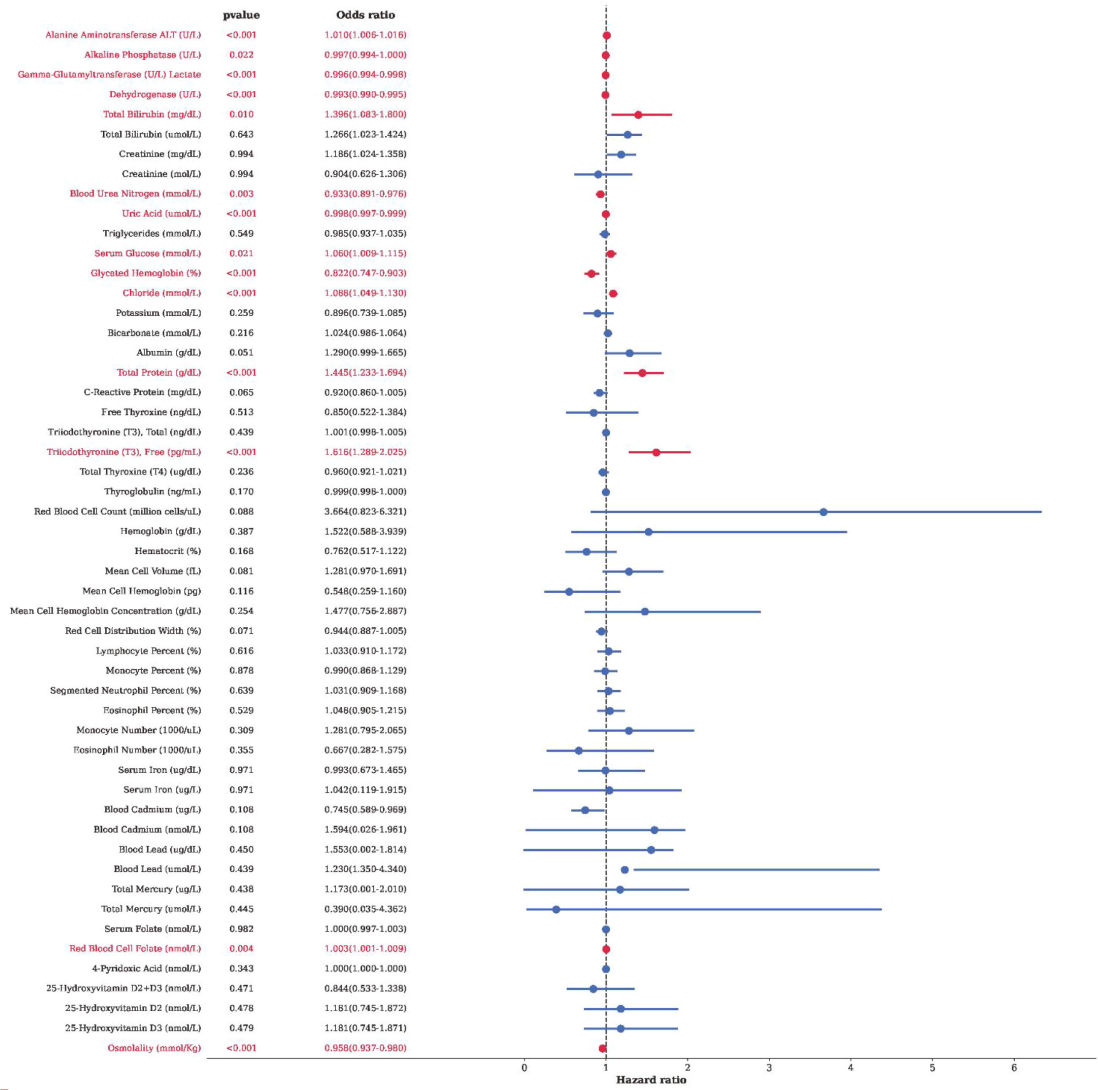
Forest plot of the associations between biochemical and immunological indicators and RA.

To further characterize blood biomarkers associated with rheumatoid arthritis, univariate and multivariable logistic regression analyses were performed (**Figure 4** and **Table 3**). Sensitivity analyses using a stricter RA definition based on self-reported RA plus DMARD use are provided in **Supplementary Tables 5**, **S6**.

**Table 3:** Association between blood biochemical indicators and RA: univariate logistic regression analysis.

Blood test indicators	OR	CI_lower	CI_upper	P_value
Alanine Aminotransferase ALT (U/L)	1.007	1.003	1.011	0.001
Aspartate Aminotransferase AST (U/L)	0.998	0.994	1.003	0.449
Alkaline Phosphatase (U/L)	0.992	0.989	0.994	0.000
Gamma-Glutamyltransferase (U/L)	0.997	0.995	0.998	0.000
Lactate Dehydrogenase (U/L)	0.989	0.987	0.991	0.000
Total Bilirubin (mg/dL)	1.393	1.142	1.700	0.001
Creatinine (mg/dL)	0.586	0.490	0.700	0.000
Blood Urea Nitrogen (mmol/L)	0.813	0.790	0.837	0.000
Uric Acid (umol/L)	0.998	0.997	0.998	0.000
Triglycerides (mmol/L)	0.940	0.903	0.978	0.002
Cholesterol (mmol/L)	1.008	0.957	1.063	0.756
Direct HDL-Cholesterol (mmol/L)	0.946	0.824	1.086	0.431
Total Cholesterol (mmol/L)	0.984	0.933	1.038	0.560
Serum Glucose (mmol/L)	0.908	0.885	0.931	0.000
Glycated Hemoglobin (%)	0.763	0.724	0.805	0.000
Total Calcium (mmol/L)	1.111	0.604	2.044	0.736
Phosphorus (mg/dL)	1.016	0.918	1.123	0.761
Chloride (mmol/L)	1.054	1.034	1.075	0.000
Potassium (mmol/L)	0.586	0.495	0.693	0.000
Sodium (mmol/L)	0.991	0.967	1.015	0.451
Bicarbonate (mmol/L)	0.969	0.945	0.994	0.014
Albumin (g/dL)	2.442	2.053	2.904	0.000
Total Protein (g/dL)	1.607	1.419	1.821	0.000
Globulin (g/dL)	1.016	0.898	1.150	0.799
C-Reactive Protein (mg/dL)	0.795	0.741	0.852	0.000
Free Thyroxine (ng/dL)	0.570	0.414	0.783	0.001
Thyroid Stimulating Hormone (uIU/mL)	0.996	0.979	1.014	0.655
Triiodothyronine (T3), Total (ng/dL)	1.015	1.012	1.017	0.000
Triiodothyronine (T3), Free (pg/mL)	3.007	2.557	3.537	0.000
Total Thyroxine (T4) (ug/dL)	0.965	0.933	0.998	0.038
Thyroglobulin (ng/mL)	0.999	0.997	1.000	0.046
Thyroglobulin Antibody (IU/mL)	1.000	0.999	1.000	0.295
Thyroid Peroxidase Antibody (IU/mL)	1.000	1.000	1.001	0.149
White Blood Cell Count (1000 cells/uL)	0.986	0.963	1.009	0.237
Red Blood Cell Count (million cells/uL)	1.667	1.483	1.873	0.000
Hemoglobin (g/dL)	1.134	1.092	1.177	0.000
Hematocrit (%)	1.043	1.030	1.058	0.000
Mean Cell Volume (fL)	0.974	0.964	0.985	0.000
Mean Cell Hemoglobin (pg)	0.959	0.935	0.985	0.002
Mean Cell Hemoglobin Concentration (g/dL)	1.089	1.026	1.157	0.005
Red Cell Distribution Width (%)	0.828	0.790	0.867	0.000
Platelet Count (1000 cells/uL)	1.000	1.000	1.001	0.292
Mean Platelet Volume (fL)	1.043	0.975	1.116	0.224
Lymphocyte Percent (%)	1.018	1.011	1.024	0.000
Monocyte Percent (%)	0.965	0.943	0.987	0.002
Segmented Neutrophil Percent (%)	0.991	0.985	0.997	0.002
Eosinophil Percent (%)	0.971	0.948	0.995	0.020
Basophil Percent (%)	0.946	0.843	1.062	0.344
Lymphocyte Number (1000/uL)	1.057	0.989	1.130	0.103
Monocyte Number (1000/uL)	0.585	0.437	0.784	0.000
Segmented Neutrophil Number (1000 cells/uL)	0.974	0.945	1.004	0.086
Eosinophil Number (1000/uL)	0.651	0.482	0.881	0.005
Basophil Number (1000 cells/uL)	0.502	0.188	1.342	0.169
Serum Iron (ug/dL)	1.004	1.002	1.005	0.000
Serum Iron (ug/L)	1.021	1.011	1.030	0.000
Blood Cadmium (ug/L)	0.842	0.771	0.918	0.000
Blood Cadmium (nmol/L)	0.981	0.971	0.990	0.000
Blood Lead (ug/dL)	0.868	0.836	0.902	0.000
Total Mercury (ug/L)	1.065	1.030	1.101	0.000
Inorganic Mercury (ug/L)	0.916	0.681	1.231	0.560
Serum Folate (nmol/L)	0.992	0.990	0.994	0.000
Red Blood Cell Folate (nmol/L)	0.999	0.999	1.000	0.000
Pyridoxal 5’-Phosphate (nmol/L)	1.000	0.999	1.001	0.848
4-Pyridoxic Acid (nmol/L)	1.000	1.000	1.000	0.000
25-Hydroxyvitamin D2+D3 (nmol/L)	0.995	0.993	0.998	0.000
25-Hydroxyvitamin D2 (nmol/L)	0.985	0.979	0.990	0.000
25-Hydroxyvitamin D3 (nmol/L)	0.997	0.995	1.000	0.024
Osmolality (mmol/Kg)	0.943	0.932	0.954	0.000

In univariate analyses, several metabolic, hepatic, and hematologic markers were statistically associated with rheumatoid arthritis. Alanine aminotransferase showed a positive association, whereas gamma-glutamyltransferase and lactate dehydrogenase were inversely associated. Serum uric acid and free triiodothyronine were also significantly associated with rheumatoid arthritis status (*P* < 0.05). In addition, multiple erythroid and nutritional markers, including red blood cell count, hemoglobin, albumin, and globulin, demonstrated significant associations in univariate models.

In multivariable analyses adjusting for potential confounders, free triiodothyronine, glycated hemoglobin, red blood cell folate, osmolality, and alanine aminotransferase remained independently associated with rheumatoid arthritis.

Overall, these results indicate that rheumatoid arthritis is accompanied by a broad pattern of concurrent alterations in metabolic, hepatic, hematologic, and micronutrient-related biomarkers. Given the cross-sectional nature of the analysis, these associations should be interpreted as descriptive and exploratory rather than indicative of causal or mechanistic relationships.

### 3.5 Intersection and mediation analysis

**Figure 5:**
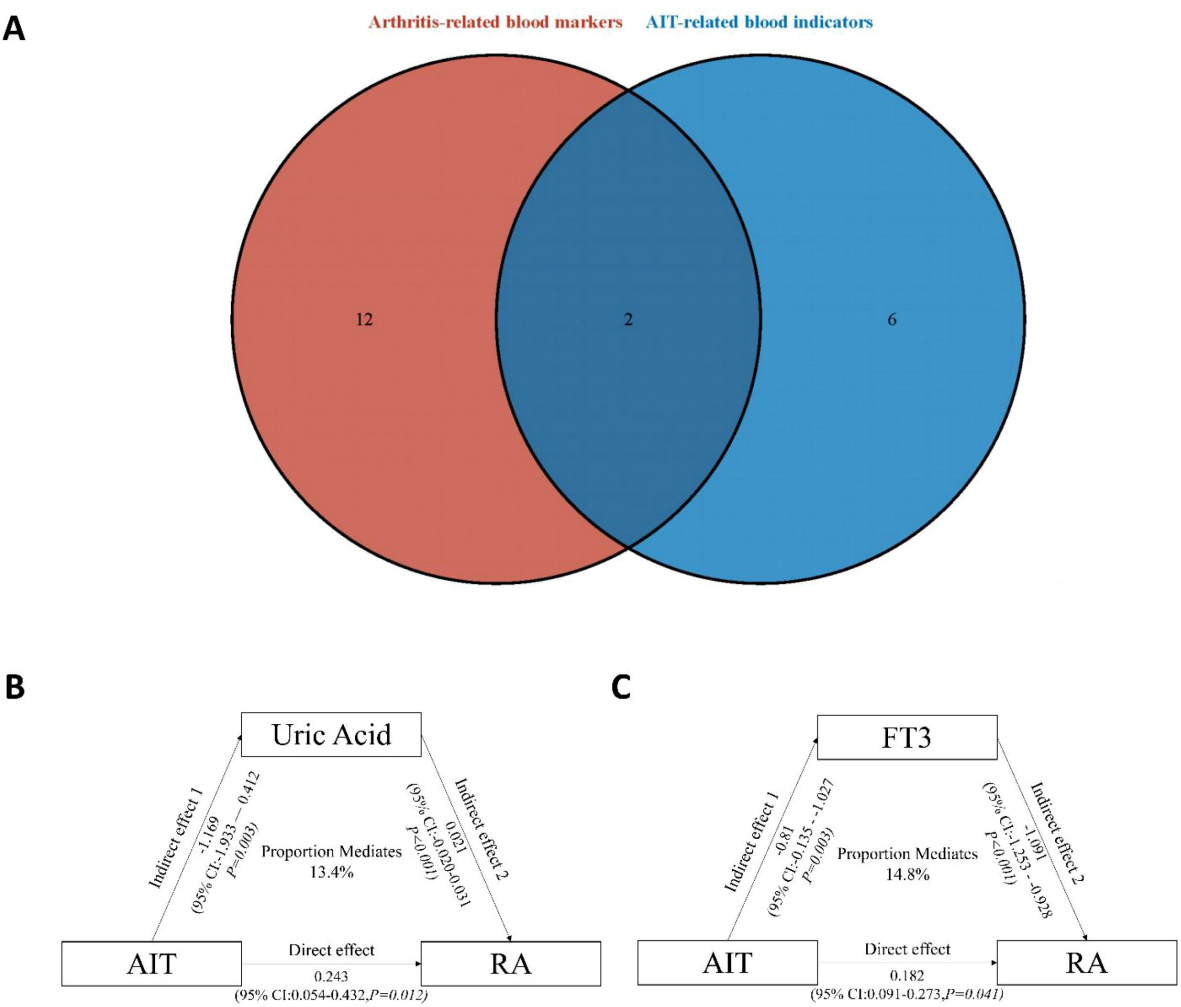
Overlap and mediation analysis of shared blood markers between AIT and RA. **(A)** Venn diagram showing the overlap between blood indicators significantly associated with AIT (blue) and those associated with RA (red). **(B)** Mediation analysis using uric acid as a mediator in the association between AIT and RA. **(C)** Mediation analysis using FT3 as a mediator.

To explore whether shared metabolic or endocrine markers were statistically related to the observed association between AIT and RA, exploratory mediation analyses were performed using uric acid and FT3 as candidate intermediates in both the NHANES cohort and an independent external validation cohort (**Figure 5**; **Table 4**). Given the cross-sectional design, these analyses were intended to provide an exploratory statistical decomposition of the observed associations rather than to establish causal, directional, or mechanistic pathways.

**Table 4:** External validation of mediation effects.

Mediator	Effect	NHANES (n=5,715)	External validation (n=200)
Uric Acid	Total Effect	0.243 (0.054, 0.432)*	0.054 (0.0072, 0.185)*
	Direct Effect (ADE)	0.213 (0.018, 0.408)*	0.035 (0.0087, 0.161)*
	Indirect Effect (ACME)	0.038	0.018
	Proportion Mediated	13.40%	33.60%
Free T3	Total Effect	0.182 (0.091, 0.273)*	0.059 (-0.070, 0.184)
	Direct Effect (ADE)	0.142 (0.033, 0.251)*	0.057 (-0.070, 0.187)
	Indirect Effect (ACME)	0.048	0.002
	Proportion Mediated	14.80%	4.10%

ACME , Average Causal Mediation Effect; ADE , Average Direct Effect.

*P < 0.05

When uric acid was evaluated as the candidate mediator, a statistically significant total effect of AIT on RA was observed in the external validation cohort (effect estimate = 0.054, 95% CI: 0.007–0.185), with a corresponding indirect effect of 0.018, yielding a mediation proportion of 33.6%. This pattern was directionally similar to that observed in the NHANES cohort, in which uric acid accounted for 13.4% of the total association. However, the magnitude of the mediated proportion differed between cohorts, and therefore these findings should be interpreted cautiously. Overall, the repeated observation of a non-zero indirect effect suggests that uric acid may represent a shared metabolic correlate of the AIT–RA association, rather than evidence of a strictly unidirectional mediating mechanism.

In contrast, mediation analyses involving FT3 showed limited consistency across cohorts. In the external validation cohort, neither the total effect nor the indirect effect reached statistical significance (total effect estimate = 0.059, 95% CI: −0.070 to 0.184; indirect effect = 0.002; mediation proportion = 4.1%). These findings differed from those in NHANES, in which a modest mediation proportion (14.8%) was observed. Such discrepancies may reflect differences in sample size, population structure, disease ascertainment, or measurement variability, and suggest that FT3-related mediation signals are less stable across datasets.

Because temporal ordering could not be established in this cross-sectional framework, additional reverse-direction mediation analyses (RA → mediator → AIT) were performed as exploratory assessments of association symmetry, and the results are presented in **Supplementary Table 7**. In addition, E-value sensitivity analyses were conducted to quantify the potential vulnerability of the mediation-related findings to unmeasured confounding, with results provided in **Supplementary Table 8**. Taken together, these supplementary analyses further support interpreting the mediation-related findings as descriptive and hypothesis-generating.

### 3.6 Discriminative model construction and performance evaluation

**Figure 6:**
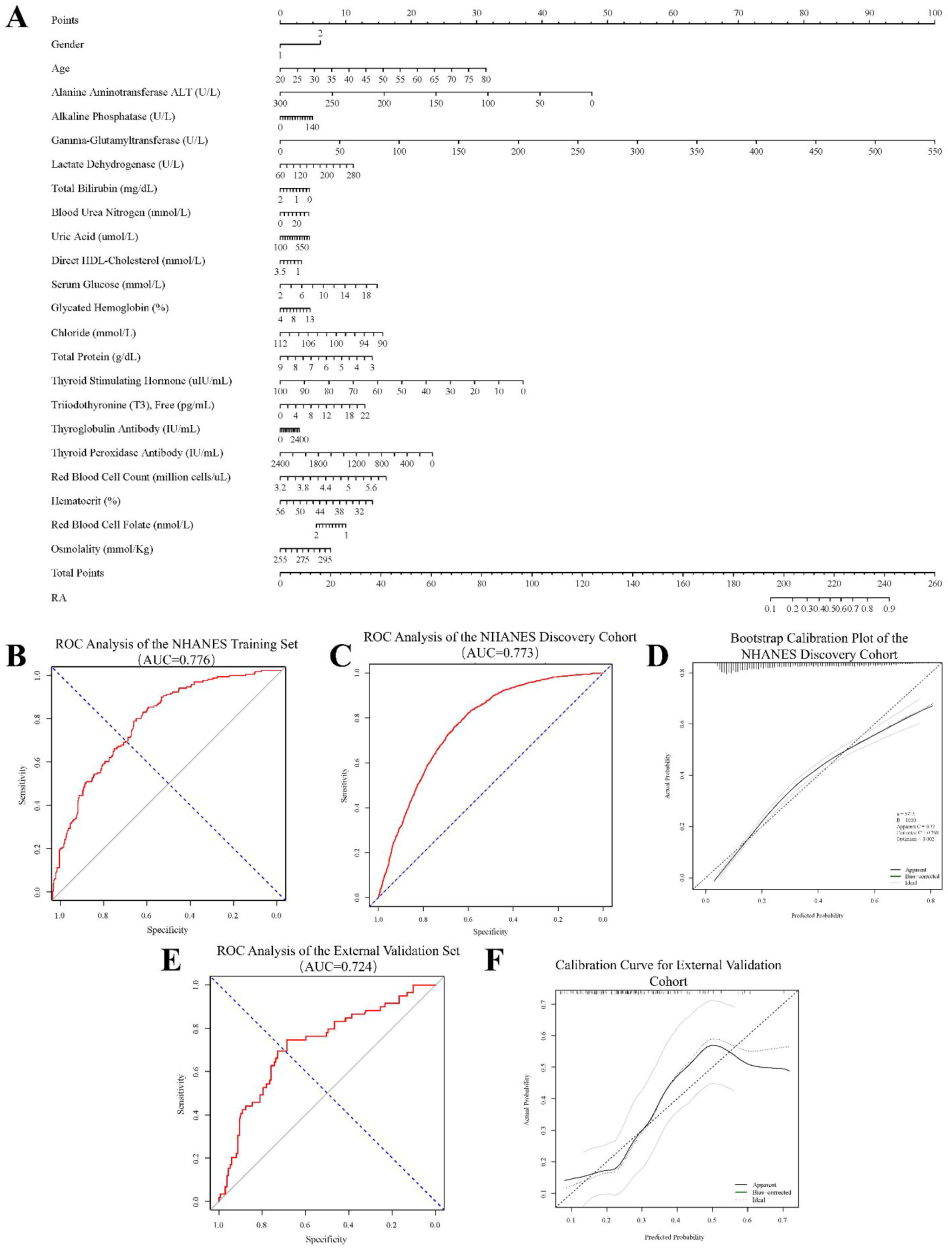
Construction and external validation of the rheumatoid arthritis prediction model among individuals with autoimmune thyroiditis. **(A)** Nomogram depicting the multivariable logistic regression model used to estimate rheumatoid arthritis (RA) risk in patients with autoimmune thyroiditis (AIT), with higher point allocations indicating stronger predictive contributions. **(B)** Receiver operating characteristic (ROC) curve demonstrating discriminative performance of the model in the NHANES discovery cohort (AUC = 0.776). **(C)** External validation ROC curve in an independent clinical cohort (AUC = 0.724), indicating preserved predictive capability in real-world settings. **(D)** Calibration plot for the external validation cohort showing good agreement between predicted and observed RA probabilities, supporting satisfactory model calibration. **(E)** ROC curve for the external validation cohort. **(F)** Calibration curve for the external validation cohort showing agreement between predicted and observed RA probabilities, supporting satisfactory model calibration.

A multivariable logistic regression model was constructed based on predictors identified in the NHANES cohort, and a nomogram was developed to visualize the relative contribution of these variables to rheumatoid arthritis (RA) status among individuals with autoimmune thyroiditis (AIT) (**Figure 6A**).

In the NHANES discovery cohort, the model demonstrated acceptable discriminative performance, with an area under the receiver operating characteristic curve (AUC) of 0.776 (**Figure 6B**). When applied to an independent hospital-based validation cohort, the model maintained moderate discrimination (AUC = 0.724; **Figure 6E**). Calibration analyses suggested reasonable agreement between predicted and observed probabilities in both the discovery and validation cohorts (**Figure 6**D and **Figure 6F**).

Taken together, these findings indicate that the proposed nomogram provides stable discrimination and calibration for RA status across datasets. Given the cross-sectional design and the limited size of the external validation cohort, the model should be interpreted as a statistical risk stratification tool rather than a predictor of incident RA. Further evaluation in larger, population-based prospective studies is required before potential clinical application.

## 4 Discussion

In this population-based cross-sectional study, we observed a significant association between autoimmune thyroiditis (AIT) and rheumatoid arthritis (RA) in a nationally representative sample, with consistent findings observed in an independent clinical cohort. Although causal inference is precluded by the cross-sectional design, the concordant associations across two distinct datasets suggest a non-random coexistence of thyroid and joint autoimmunity. In addition, uric acid and free triiodothyronine (FT3) were identified as statistically associated intermediates linking AIT and RA. Notably, while the mediation pattern for uric acid was observed in both datasets, the mediation effect of FT3 did not reach statistical significance in the external validation cohort. These findings should therefore be interpreted as exploratory and hypothesis-generating, pointing toward potential metabolic and endocrine correlates of autoimmune disease clustering that warrant further evaluation in longitudinal and mechanistic studies.

One notable finding of our study is the identification of uric acid as a statistically associated intermediate between thyroid autoimmunity and joint inflammation. Although traditionally associated with gout and renal function, uric acid has increasingly been recognized as being involved in immune activation and oxidative stress (12). Elevated uric acid levels have been linked to inflammasome activation, whereas lower levels, as observed in autoimmune conditions, may reflect alterations in purine metabolism and systemic inflammatory states (13). In our analysis, reduced uric acid levels were independently associated with both conditions and accounted for approximately 13.4% of the observed association between autoimmune thyroiditis and rheumatoid arthritis. This finding is consistent with an emerging body of literature suggesting that uric acid may function not only as a metabolic waste product but also as an indicator of immune-related biological processes (8, 14, 15).

The association involving free triiodothyronine, the biologically active form of thyroid hormone, represents another notable observation in our study (16). While its involvement in thyroid function is well established, emerging research has increasingly implicated thyroid hormones in modulating innate and adaptive immunity. Experimental studies have suggested that triiodothyronine may be associated with a shift toward pro-inflammatory monocyte/macrophage phenotypes and with reduced cardiac regenerative capacity, with broadly similar patterns observed in zebrafish cardiac regeneration models (17, 18). In our analysis, lower levels of free triiodothyronine were statistically associated with both autoimmune thyroiditis and rheumatoid arthritis, and contributed modestly to the observed association between the two conditions. This observation suggests that alterations in endocrine homeostasis may be linked to autoimmune susceptibility, although the direction and underlying mechanisms remain to be clarified. Moreover, thyroid hormone signaling has been shown to influence T cell differentiation, cytokine production, and macrophage activation, processes that are central to immune regulation and autoimmune-related inflammation (19).

Importantly, the observed mediating effects were partial rather than complete, suggesting that additional, unmeasured factors may contribute to the observed association between the two diseases. This underscores the complexity of immune regulation and the multifactorial nature of autoimmune disease manifestations. Factors such as genetic susceptibility, microbiome alterations, environmental exposures, and epigenetic modifications likely interact in a complex network that cannot be fully captured by a single mediator model. Future studies incorporating longitudinal data, genetic profiling, and multi-omics approaches may help disentangle these intricate relationships.

Another contribution of this study is the development of a statistical risk stratification model to characterize rheumatoid arthritis status among individuals with thyroid autoimmunity. The model demonstrated acceptable discriminative performance, with an area under the receiver operating characteristic curve exceeding 0.77, and showed stable performance across ten-fold cross-validation. The nomogram visualization improves model interpretability by providing an intuitive representation of the relative contribution of individual predictors. Notably, the key predictors included in the model—such as free triiodothyronine, thyroid antibodies, and hematologic indices—are routinely measured laboratory parameters, which may facilitate further evaluation of the model in future clinical and population-based studies.

This study provides robust, population-based evidence supporting a significant association between autoimmune thyroiditis and rheumatoid arthritis, which was further strengthened through external clinical validation. Although causality cannot be inferred from the present cross-sectional design, the exploratory mediation findings suggest that metabolic and endocrine alterations—particularly involving uric acid and thyroid hormone regulation—may partially contribute to autoimmune clustering. These insights align with the broader concept that autoimmune diseases represent interconnected phenotypes within systemic immune dysregulation. Future longitudinal studies and mechanistic investigations are warranted to clarify temporal pathways and to determine whether early recognition of thyroid autoimmunity may help identify individuals at heightened risk for joint involvement, ultimately informing strategies for integrated autoimmune disease prevention and management.

## 5 Conclusion

This study demonstrates a statistically significant association between autoimmune thyroiditis and rheumatoid arthritis in a nationally representative population, with consistent findings observed in an independent clinical cohort. Although causal relationships cannot be inferred from the cross-sectional design, exploratory mediation analyses suggest that metabolic and endocrine alterations—particularly those involving uric acid and thyroid hormone–related markers—may be associated with the observed coexistence of these autoimmune conditions. These findings support the concept that autoimmune diseases may share overlapping metabolic and immunological contexts rather than occurring as isolated entities. Further longitudinal and mechanistic studies are required to clarify temporal relationships and underlying biological pathways. Such investigations may help to better characterize autoimmune clustering and inform future research on integrated approaches to autoimmune disease assessment.

## Data Availability

The original contributions presented in the study are included in the article/**Supplementary Material**. Further inquiries can be directed to the corresponding author.
