# Supplementary material for: Association between autoimmune thyroiditis and rheumatoid arthritis: a cross-sectional risk stratification study
Source: Front Endocrinol (Lausanne). 2026 Apr 1;17:1764735. doi: 10.3389/fendo.2026.1764735 (PMC13079147; doi:10.3389/fendo.2026.1764735)
Supplement: Supplementary file 1 [file DataSheet1.docx]

Supplementary Materials

Figure S1. The flowchart of participant selection for the external validation cohort





| Table S1. Baseline characteristics of the external cohort | | | | |
| --- | --- | --- | --- | --- |
|  | Overall  ( n=200 ) | Non-AIT  ( n=100 ) | AIT  ( n=100 ) | P |
| Age (mean (SD)) | 45.73 (13.66) | 46.50 (13.74) | 44.96 (13.61) | 0.427 |
| Height (mean (SD)) | 161.06 (7.77) | 161.81 (7.98) | 160.30 (7.53) | 0.171 |
| Weight (mean (SD)) | 62.00 (10.88) | 61.77 (10.98) | 62.23 (10.82) | 0.768 |
| BMI (mean (SD)) | 23.86 (3.53) | 23.52 (3.35) | 24.20 (3.68) | 0.179 |
| WC (mean (SD)) | 93.94 (8.95) | 93.62 (8.92) | 94.25 (9.02) | 0.623 |
| TSH (median [IQR]) | 1.50 [1.08, 2.36] | 1.34 [1.06, 1.90] | 1.72 [1.20, 2.59] | 0.008 |
| FT3 (mean (SD)) | 4.40 (0.58) | 4.40 (0.52) | 4.39 (0.64) | 0.955 |
| FT4 (mean (SD)) | 12.29 (1.80) | 12.09 (1.83) | 12.49 (1.75) | 0.114 |
| TPOAb (median [IQR]) | 3.00 [3.00, 58.24] | 3.00 [3.00, 3.00] | 59.33 [7.69, 460.89] | <0.001 |
| TgAb (median [IQR]) | 20.87 [15.38, 142.75] | 15.85 [14.05, 18.28] | 144.50 [39.07, 375.00] | <0.001 |
| UA (mean (SD)) | 324.39 (86.94) | 321.08 (80.29) | 327.80 (93.59) | 0.589 |
| Glucose (mean (SD)) | 5.13 (0.95) | 5.12 (0.93) | 5.14 (0.98) | 0.87 |
| TG (median [IQR]) | 1.08 [0.83, 1.58] | 1.04 [0.82, 1.46] | 1.15 [0.86, 1.62] | 0.322 |
| TC (mean (SD)) | 4.78 (1.04) | 4.81 (1.00) | 4.76 (1.08) | 0.755 |
| HDL (mean (SD)) | 1.43 (0.32) | 1.45 (0.35) | 1.41 (0.29) | 0.428 |
| LDL (mean (SD)) | 2.79 (0.72) | 2.80 (0.72) | 2.78 (0.72) | 0.856 |
| ALT (median [IQR]) | 19.30 [14.00, 27.73] | 19.00 [14.00, 25.00] | 20.50 [14.00, 29.88] | 0.447 |
| AST (median [IQR]) | 21.00 [18.00, 25.78] | 21.00 [18.00, 25.12] | 21.00 [18.00, 26.70] | 0.849 |
| Cr (mean (SD)) | 62.64 (14.09) | 63.76 (16.23) | 61.48 (11.43) | 0.256 |
| BUN (mean (SD)) | 5.13 (1.44) | 5.09 (1.53) | 5.17 (1.35) | 0.723 |
| ALB (mean (SD)) | 44.59 (2.78) | 44.37 (2.84) | 44.82 (2.71) | 0.252 |
| Hb (mean (SD)) | 135.92 (13.66) | 137.10 (14.68) | 134.71 (12.49) | 0.22 |
| RBC (mean (SD)) | 4.63 (0.44) | 4.65 (0.43) | 4.61 (0.46) | 0.474 |
| HCT (mean (SD)) | 41.29 (4.82) | 41.57 (5.80) | 41.02 (3.56) | 0.425 |
| CRP (median [IQR]) | 0.21 [0.13, 0.33] | 0.18 [0.13, 0.27] | 0.22 [0.14, 0.36] | 0.014 |
| Sex = Male (%) | 44 (22.0) | 28 (28.0) | 16 (16.0) | 0.06 |
| RA = Yes (%) | 61 (30.5) | 28 (28.0) | 33 (33.0) | 0.539 |
| Diabetes = Yes (%) | 16 (8.0) | 8 (8.0) | 8 (8.0) | 1 |
| Hypertension = Yes (%) | 30 (15.0) | 17 (17.0) | 13 (13.0) | 0.552 |
| Gout = Yes (%) | 1 (0.5) | 1 (1.0) | 0 (0.0) | 1 |
| CHD_HF = Yes (%) | 1 (0.5) | 0 (0.0) | 1 (1.0) | 1 |
| CKD = Yes (%) | 1 (0.5) | 1 (1.0) | 0 (0.0) | 1 |

A total of 200 participants were included in the external validation cohort, comprising 100 patients with autoimmune thyroiditis (AIT) and 100 non-AIT controls. As shown in Supplementary Table S1, there were no significant differences between the two groups in terms of age, sex distribution, anthropometric measurements (BMI and WC), thyroid-stimulating hormone (TSH), FT3, FT4, and most metabolic biomarkers (all P > 0.05).

In contrast, thyroid autoantibody levels were significantly higher in the AIT group, including TPOAb and TgAb (both P < 0.001), consistent with clinical diagnostic expectations. Additionally, the AIT group showed slightly elevated CRP levels compared with controls (P = 0.014), suggesting a higher degree of systemic inflammation.

Comorbidities including diabetes, hypertension, hyperuricemia-related gout, coronary heart disease/heart failure, and chronic kidney disease showed no significant inter-group differences (all P > 0.05), indicating well-balanced health profiles between groups aside from thyroid autoimmunity-related biomarkers.

Overall, the comparable demographic and clinical profiles support the suitability of this external cohort for evaluating the generalizability and calibration performance of the prediction model.

| Table S2. Comparison of baseline characteristics between the external validation cohort and the NHANES database cohort | | | | |
| --- | --- | --- | --- | --- |
| Variable | External validation cohort  (n = 196) | Database cohort  (n = 5,715) | P |  |
| Age (mean (SD)) | 45.73 (13.66) | 50.48 (22.00) | <0.001 |  |
| TSH (median (IQR)) | 1.50 [1.08, 2.36] | 1.71 (1.09) | 0.384 |  |
| FT3 (mean (SD)) | 4.40 (0.58) | 3.12 (0.37) | <0.001 |  |
| FT4 (mean (SD)) | 12.29 (1.80) | 10.30 (1.93) | <0.001 |  |
| TPOAb (median (IQR)) | 3.00 [3.00, 58.24] | 17.30 (80.00) | 0.183 |  |
| TgAb (median (IQR)) | 20.87 [15.38, 142.75] | 2.69 (15.71) | <0.001 |  |
| UA (median (IQR)) | 324.39 (86.94) | 324.03 (87.34) | 0.954 |  |
| Glucose (mean (SD)) | 5.13 (0.95) | 5.25 (0.82) | 0.087 |  |
| TG (median [IQR]) | 1.08 [0.83, 1.58] | 1.54 (0.93) | <0.001 |  |
| TC (mean (SD)) | 4.78 (1.04) | 5.01 (1.07) | 0.002 |  |
| HDL (mean (SD)) | 1.43 (0.32) | 1.30 (0.41) | <0.001 |  |
| ALT (median [IQR]) | 19.30 [14.00, 27.73] | 21.94 (8.15) | 0.030 |  |
| AST (median [IQR]) | 21.00 [18.00, 25.78] | 23.67 (5.93) | <0.001 |  |
| Cr (mean (SD)) | 62.64 (14.09) | 75.71 (19.39) | <0.001 |  |
| BUN (mean (SD)) | 5.13 (1.44) | 4.53 (1.59) | <0.001 |  |
| ALB (mean (SD)) | 44.59 (2.78) | 4.20 (0.30) | <0.001 |  |
| Hb (mean (SD)) | 135.92 (13.66) | 14.20 (1.55) | <0.001 |  |
| RBC (mean (SD)) | 4.63 (0.44) | 4.65 (0.51) | 0.537 |  |
| HCT (mean (SD)) | 41.29 (4.82) | 41.35 (4.45) | 0.854 |  |
| CRP (median [IQR]) | 0.21 [0.13, 0.33] | 0.25 (0.28) | 0.045 |  |
| Sex (Male) (%)) | 44 (22.0) | 2851 (49.9) | <0.001 |  |
| RA = Yes (%) | 61 (30.5) | 1664 (29.1) | 0.672 |  |
| Diabetes = Yes (%) | 16 (8.0) | 814 (14.2) | 0.012 |  |
| Hypertension = Yes (%) | 30 (15.0) | 2063 (36.1) | <0.001 |  |
| Gout = Yes (%) | 1 (0.5) | 296 (5.2) | 0.003 |  |

| Table S3. Sensitivity Analysis Excluding Patients with Gout | | | | |
| --- | --- | --- | --- | --- |
| **Parameter** | **Main Analysis (n=5,715)** | **Sensitivity Analysis (n=5,419, gout excluded)** | **Interpretation** |  |
| **Sample Characteristics** | | | |  |
| Total sample size, n | 5,715 | 5,419 | Retained 94.8% |  |
| AIT prevalence, % | 9.5 | 14.8 | Comparable |  |
| RA prevalence, % | 28.7 | 27.4 | Comparable |  |
| **Primary Finding: AIT-RA Association** | | | |  |
| RA prevalence in AIT, % | 33.2 | 31.9 | Consistent |  |
| RA prevalence in non-AIT, % | 28.7 | 26.6 | Consistent |  |
| OR (95% CI) | 1.236 (1.024-1.492) | 1.294 (1.100-1.522) | Association maintained |  |
| P-value | 0.027 | 0.002 | Remained significant |  |
| **Key Mediating Biomarkers** | | | |  |
| Uric acid in AIT, μmol/L | 318.8 ± 81.4 | 316.0 ± 81.1 | Lower in AIT |  |
| Uric acid in non-AIT, μmol/L | 330.5 ± 83.1 | 327.0 ± 83.5 | Pattern preserved |  |
| P-value for uric acid | 0.003 | 0.001 | Remained significant |  |
| Free T3 in AIT, pg/mL | Not reported | 3.12 ± 0.86 | Lower in AIT |  |
| Free T3 in non-AIT, pg/mL | Not reported | 3.19 ± 0.56 | Pattern preserved |  |
| P-value for free T3 | <0.001 | 0.004 | Remained significant |  |

| Table S4. Sensitivity Analysis Using Alternative Definitions of AIT | | | | |
| --- | --- | --- | --- | --- |
| **AIT Definition** | **N** | **OR** | **95% CI** | **P value** |
| **Original definition (antibody positivity only)** | 545 | 1.236 | 1.024-1.492 | 0.027* |
| **Strict definition (antibody positivity + abnormal TSH)** | 207 | 1.119 | 0.830-1.509 | 0.461 |
| **Antibody positivity with normal TSH** | 631 | 1.225 | 1.027-1.462 | 0.024* |
| **TPOAb ≥ 500 IU/mL** | 65 | 1.0054 | 0.892-1.528 | 0.0909 |

| Table S5. Availability of DMARD Data | |
| --- | --- |
| **Item** | **Value** |
| **Total participants** | 5715 |
| **Has_DMARD non-missing** | 619 |
| **Has_DMARD =1** | 23 |
| **RA =1** | 1664 |
| **RA_strict = 1** | 19 |

| Table S6. Sensitivity Analysis Using a Strict RA Definition (RA + DMARD) | | | | | |
| --- | --- | --- | --- | --- | --- |
| **Outcome_definition** | **OR** | **CI_Lower** | **CI_Upper** | **P_value** | **Events** |
| **Original outcome: self-reported RA** | 0.89 | 0.73 | 1.10 | 0.30 | 1664 |
| **Strict outcome: RA + DMARD** | 0.37 | 0.05 | 2.82 | 0.34 | 19 |

Table S7. Reverse Mediation Analysis: RA → Mediator → AIT

| **Mediator** | **Direction of Analysis** | **Indirect Effect** | **95% CI** | **Mediation Proportion** | **Significance** |
| --- | --- | --- | --- | --- | --- |
| UA | RA → UA → AIT | −0.0315 | (−0.0550, 0.0111) | 14.9% | Significant* |
| FT3 | RA → FT3 → AIT | 0.0722 | (−0.0055, 0.1751) | 34.1% | Not significant |

| Table S8. E-value Sensitivity Analysis for Mediation-Related Associations | | | | | | |
| --- | --- | --- | --- | --- | --- | --- |
| **Association** | **OR** | **CI_Lower** | **CI_Upper** | **P value** | **E value (point estimate)** | **E value (CI limit)** |
| **Total effect AIT->RA** | 0.89 | 0.73 | 1.10 | 0.30 | 1.48 | 1.44 |
| **Mediator-outcome UA->RA (adj AIT)** | 1.00 | 1.00 | 1.00 | 0.01 | 1.03 | 1.02 |
| **Mediator-outcome FT3->RA (adj AIT)** | 0.93 | 0.80 | 1.08 | 0.35 | 1.36 | 1.38 |
